# Moderate exercise may attenuate some aspects of immunosenescence

**DOI:** 10.1186/1471-2318-4-8

**Published:** 2004-09-29

**Authors:** Nadzieja Drela, Ewa Kozdron, Piotr Szczypiorski

**Affiliations:** 1 Department of Immunology, Warsaw University, Warsaw, Poland; 2 Department of Recreation, Academy of Physical Education, Warsaw, Poland; 3 Department of Sports Medicine, Academy of Physical Education, Warsaw, Poland

## Abstract

**Background:**

Immunosenescence is related to the deterioration of many immune functions, which may be manifested in increased susceptibility to infection, cancer, and autoimmunity. Lifestyle factors, such as diet or physical activity, may influence the senescence of the immune system. It is widely accepted that moderate physical activity may cause beneficial effects for physical and psychological health as well as for the immune system activity in aged people.

**Methods:**

Thirty elderly women aged 62 to 86 were subjected to a two-years authorized physical activity program. Peripheral blood lymphocytes distribution and the production of cytokines involved in the immune response development and regulation (IL-2, IL-4 and IFN-γ) were investigated. The same parameters were evaluated in two control groups of women: a sedentary group of 12 elderly women selected for the second round of the physical activity program and in a group of 20 sedentary young women. Flow cytometry methods were used for the examination of surface markers on peripheral blood lymphocytes and intracellular cytokines expression.

**Results:**

The distribution of the main lymphocytes subpopulations in the peripheral blood of elderly women did not show changes after long-term moderate physical training. The percentage of lymphocytes expressing intracellular IL-2 was higher in the group of women attending 2-years physical activity program than in the control group of elderly sedentary women, and it was similar to the value estimated in the group of young sedentary women. There was no difference in the intracellular expression of IL-4 and IFN-γ between the active and elderly sedentary women.

**Conclusions:**

Our results suggest that moderate, long-term physical activity in elderly women may increase the production of IL-2, an important regulator of the immune response. This may help ameliorate immunosenescence in these women.

## Background

The decline in immune function associated with ageing increases the risk for infectious diseases, autoimmune disorders and tumors occurrence. The scientific interest of many laboratories is focused on the ageing of the immune system and on agents, which may retardate this process. The most frequently mentioned features of immunosenescence characterized by lymphocytes surface markers expression are: shift from naive CD45 RA^+ ^to memory CD45 RO^+ ^T cells, increased level of CD4^+ ^(mainly in peripheral tissues), increased level of NK cells, and decreased level of CD8^+ ^and B cells [1-4]. Changes in the distribution of T cell subpopulations in the blood are considered rather not significant (with the exception of naïve/memory T cells). However data are still inconsistent and may be caused by the criteria used to select healthy old individuals and by differences between human populations depending on race or geographical region [5-8].

Changes in cytokine production are manifested by the shift from Th1 to Th2-type cytokine production and increased level of proinflammatory cytokines (TNF-α, IL-1β, IL-6) [9-12]. The decrease of T cells responsiveness to in vitro stimulation may result from the reduction of IL-2 secretion and IL-2R α chain expression [13]. The most influenced by advancing age is the cell-mediated immunity, which is directly attributed to age-associated involution of thymus gland [14,15]. Recently, the interest of many groups and medical centers is focused on the benefits of regular exercise by older people. These benefits include enhanced cardiovascular fitness, retention of muscle mass, reduction of risk factors associated with many life-threatening diseases. There is still poor documentation of the possibility to alter the activity of the immune system as a consequence of exercise in older population. It has been shown that natural immunity is strongly influenced by physical exercise [16,17]. There is some evidence that the plasma levels of various, mainly pro-inflammatory cytokines increase in response to strenuous exercise [18]. A new area of research points on the relationship between certain lifestyle factors (diet, physical activity) and immune senescence. The group of 30 elderly women was selected to participate in the program of physical activity supervised by the Academy of Physical Education in Warsaw (Poland). The purpose of this program was to evaluate the modulation of physiological and psychological parameters by moderate long-term training. The beneficial results of moderate training on different body functions created the idea to check selected parameters, which characterize the activity of the immune system. Blood lymphocyte subpopulations distribution and intracellular expression of cytokines, which are central regulators of the immune responses (IL-2, IL-4 and IFN-γ), in activated blood lymphocytes have been examined. The aim of these investigations was to answer the main question related to the effect of exercise in older people: can long-term, moderate exercise, attenuate changes attributed to aged immune system? Comparative studies have been performed between the group of elderly exercising women and two control groups: elderly women selected for the new round of physical activity program and young sedentary women.

## Methods

### Characteristics of the group

The group investigated consisted of 30 women aged 62 to 86 (mean age 73.2) with no contraindication of consistent workout found. This group of surveyed women had never attended regular organized exercise classes previously. The group attended 2-years authorized physical activity program aimed at taking into account the basic requirements for health regarding prophylaxis of diseases of the circulatory and respiratory systems and the kinetic system. The exercises were done twice a week for 50 minutes during 10 months of the year. The annual cycle has been concluded with a two-week trip with a profile of recreation and tourism. Each workout session was similar in nature with equal time for each exercise. The initial part (10 minutes) consisted of exercises preparing the body for physical exertion simply called "warm up" preferred to be done in higher positions. The main part (30 minutes) of different intensity consisted of: a/ workout while marching and standing – aimed at improving the condition of the circulatory and respiratory system, b/ workout in low positions – in squat, on hands and knees, lying on your side, in prone position – which intended to strengthen the kinetic system. The exercises finished with a 5 minutes "cool down" and relaxation exercises done in low positions such as lying and squat positions. The general rate of exercises was stimulated with music in character and rhythm, which was adapted to the age and abilities of those who participated in exercises and it, was from 100 to 120 bpm. The intensity of exercises was regulated additionally by the number of repetitions of particular exercises and series, the integration of the particular movements into the entire unit or set, the choice and the method of performing breathing exercises. During the exercises the heart rate was monitored and registered by a Polar Sport Tester. The pulse rate was not higher than 80% of the maximum load of exertion (100% of maximum load of effort during exercises = 200-age, according to the Worms rule). The work rate intensity was 60–80% of the maximum load.

Constant observation of the attendants and systematic medical examination allowed the individual immediate modifications of the load of exertion while the women were exercising. Psychophysical self-control and self-evaluation had been installed among the participants since the very first workout session based on: heart rate (this was observed before, during and after exercising), frame of mind estimation, and subjective grading of the level of difficulty of the exercises according to the Borg Scale. Participants of the exercises had been under permanent medical control. No health contraindications for attending the exercises were found during the two years observation of the participants in the workout classes. The beneficial results of the workout program have been discovered in the research works concerning: 1/ motor activity competence, 2/ mobility of joints, 3/ physical efficiency 4/ body component, 5/ post-urography, 6/ nourishment, 7/ internal evaluation, 8/ laboratory works, 9/ orthopedics, 10/ bone density evaluation, 11/ psychology [19,20].

The analysis of peripheral blood lymphocyte subpopulations and expression of intracellular cytokines were performed after 2-years physical activity program. As a control we used the group of selected 12 women (aged 60–72) starting the new round of the program (Ctrl A), and the group of 20 sedentary young healthy women 20–40 years old (Ctrl B).

### Preparation of cells

Blood was collected from donors at 08.30 – 10.00 am. PBMCs were isolated from freshly drawn heparinized blood by Lymphocyte Separation Medium (GIBCO BRL) centrifugation. Mononuclear cells from the interface were collected and washed with PBS.

### Peripheral blood lymphocyte subpopulations distribution

Blood lymphocyte subpopulations were assessed by surface markers expression. For immunofluorescence staining of human lymphocytes the Lysed Whole Blood Method was used according to Becton-Dickinson protocol. Standard set of monoclonal antibodies against surface antigens was used: Simultest™ CD3/CD8, Simultest™ CD3/CD4, Simultest™ CD3/CD19, Simultest™ CD3/CD16CD56, Simultest™ Leucogate™ (CD45/CD14) and Simultest™ Control γ_1_/γ_2a _(all reagents from Becton-Dickinson). The results are given as % of positively stained cells in the sample.

### Identification of lymphocytes expressing intracellular cytokines

PBMCs were suspended in RPMI 1640 medium with glutamax II supplemented with 1 mM sodium pyruvate, 5 × 10^-5 ^M 2-mercaptoethanol, 20 mM HEPES and 5 mcg/mL gentamicin (GIBCO BRL). For the induction of cytokines synthesis peripheral lymphocytes were activated by PMA and calcium ionophore A 23187 (Sigma) at concentrations 50 ng/mL and 250 ng/mL, respectively. Approximately 3 × 10^6 ^cells in culture medium were placed in 24-well tissue culture plates and incubated with stimulants for 14 hours at 37°C, 5% CO_2_. Monensin was used as the protein transport inhibitor and was added to the cultures for the last 5 hours incubation. Completed the incubation, the cells were harvested and tested for viability by trypan blue exclusion. For intracellular cytokines staining the IC Screen™ Intracellular Staining Kits for human IL-2, IL-4 and IFN-γ were used (Biosource Int.). Intracellular staining was performed according to the Biosource protocol. Positively stained cells were analyzed by CellQuest™ software on FACSCalibur (Becton-Dickinson). Results are presented as the percentage of cells expressing intracellular cytokines in total lymphocytes population.

### Statistical analysis

Data have been expressed as arithmetical mean and SD. Analysis of variance (ANOVA) was used to determine significant differences between control and experiments. Significance was determined at P < = 0.05.

## Results

### Phenotypes examination of peripheral blood lymphocytes

**Figure 1:**
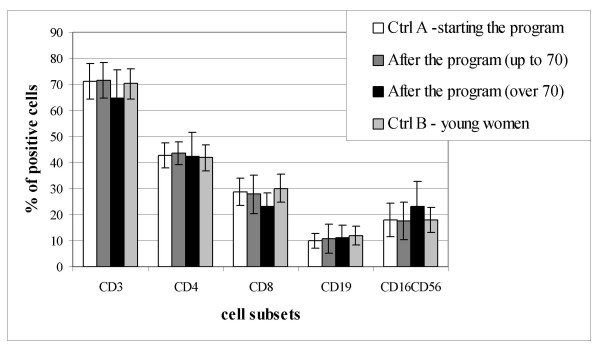
Distribution of peripheral blood lymphocyte subpopulations. Ctrl A – elderly women starting with the physical activity program. After the program (up to 70) – up to 70 years old women after the physical activity program. After the program (over 70) – over 70 years old women after the physical activity program

The percentages of lymphocytes subpopulations characteristic for exercised women were presented separately for 16 women up to 70 years old, and 14 exercised women over 70 (Fig. 1). The decrease in mature CD3^+ ^T cells with age is not a continuous process. The percentage of CD3 ^+ ^lymphocytes is rather constant until the seventh decade and decreases after this period (21, 22). The CD3 level was the purpose to present phenotypes distribution separately for women over/up 70. The distribution of the main lymphocyte subpopulations did not change among the groups. Typical changes, however not statistically significant, in lymphocyte subsets distribution in peripheral blood of women over 70 years old have been observed: decrease of the percentage of CD3^+ ^and CD8^+ ^cells, and increase in the percentage of NK cells (CD16^+^CD56^+^).

### Expression of intracellular IL-2, IL-4 and IFN-γ

**Figure 2:**
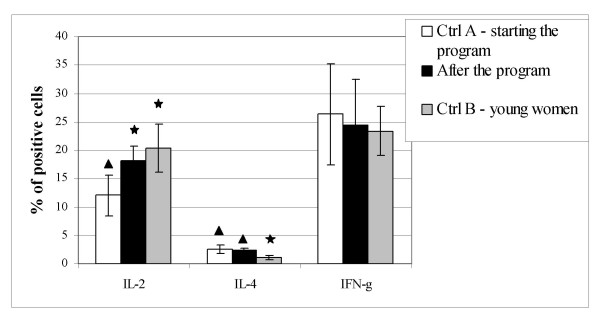
Intracellular cytokines expression in activated peripheral blood lymphocytes. Ctrl A – elderly women starting with the physical activity program. After the program – elderly exercised women. Ctrl B – young, sedentary women. Statistically significant data from Ctrl A are denoted by black stars. Statistically significant data from Ctrl B are denoted by black triangles.

**Figure 3:**
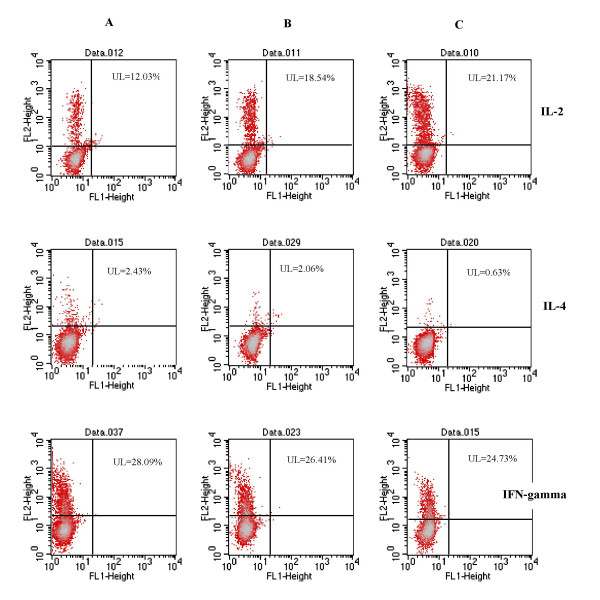
Intracellular cytokines expression in activated peripheral blood lymphocytes. A/ Ctrl A – elderly women starting with the physical activity program, B/ elderly exercised women, C/ Ctrl B – sedentary, young women. FL2 – lymphocytes positive for intracellular cytokines. Lymphocytes with intracellular cytokine expression are situated in the upper left (UL) part of the quadrant.

The percentages of in vitro activated peripheral blood lymphocytes expressing intracellular IL-2 of older exercised vs. young sedentary women were similar. Both percentages were significantly higher compared to the value obtained for older women starting the physical activity program (Fig. 2). The percentages of lymphocytes positive for IL-4 were significantly higher in older vs. young women independently on physical training. Changes in the percentages of intracellular IFN-γ expressing lymphocytes were not observed. Representative results are also presented on density plots (Fig. 3).

### The exercise intensity basing on maximum heart rate and Borg scale

The exercise intensity was evaluated basing on heart rate, assuming that 80% of effort possibilities of exercising individuals (acc. to the formula 100% = 200-age) were not exceeded. When the exercise pulse was measured during training session it was stated that the average value of HR max amounted to 105,5 +/- 6,7 bpm (90–116 bpm). At the same time the participants of training group evaluated their effort by the subjective method according to the Borg scale. The average value of this evaluation was 11,2 +/- 1,1 points (9–13 points) so it means that the participants estimated their effort as fairly light. According to Borg the results of the subjective evaluation (in points) multiplied by 10 should correspond with the heart rate measured during the effort. Thus, the exercise intensity evaluated basing on the average value of HR max (105,5 bpm) was similar to the subjective evaluation of an effort acc. to the Borg scale (11,2 points × 10 = 112 bpm).

## Discussion

Moderate physical activity may have several beneficial effects for physical and psychological health and for immune system activity in young and aged individuals. Strenuously performed exercise may cause harmful effects. The balance between beneficial and undesirable effects might be of great importance especially in older people. Exercise may mobilize NK cells, which may have originated from the liver [23]. Moderate endurance training results in increasing capacity to generate IFN-γ, but repeated exhausting exercise tends rather to down regulation of this cytokine [24]. Results of experiments performed on animal model revealed that exercised vs. control rats had greater numbers of leukocytes in the thymus, axial, and inguinal nodes. The percentage of CD4^+ ^lymphocytes increased after exercise in the thymus, spleen and blood [25]. Results of our study did not show changes in the peripheral blood lymphocyte subpopulations distribution in the group of elderly exercised women. Cytokines generate signals required for the communication among cells of the immune system. IL-2, IL-4 and IFN-γ are multifunctional cytokines involved in the development and effectors functions regulation of T, B and NK cells [26-28]. The results of our study demonstrated that the percentages of lymphocytes expressing intracellular IL-4 were higher in both groups of older women (exercised and Ctrl A) than in the group of young sedentary woman (Ctrl B). One possible explanation is the higher level of memory lymphocytes in elderly [29]. The percentage of IFN-γ positive cells did not differ significantly among groups. The percentage of lymphocytes expressing intracellular IL-2 in the group of exercised women is higher from the value obtained for older sedentary women and similar to the value characteristic for young women. Normally the level of IL-2 estimated by ELISA decreased with ageing [13]. Increase in IFN-γ production and decrease in IL-2 production have been observed in old mice [30]. IL-2 is a multifunctional cytokine essential for T cells development in the thymus and for growth in the periphery. It is involved in the maintenance of lymphocyte homeostasis [31]. IL-2 deficiency may lead/facilitate multiorgan inflammation and the formation of autoantibodies of various specificities [32]. The exercise-induced increase of the percentage of IL-2 expressing lymphocytes may sustain the activity of NK cells and the generation of cytotoxic lymphocytes. In elderly humans changes in T memory versus T naïve cells is accompanied by diminished activity in IL-2 synthesis and elevated production of IL-4 and IFN-γ. It may be possible that the effect of moderate physical activity modulate the reactivity of T cells and other cells of the immune system despite physiological changes related to ageing. Recent data demonstrate that in vitro treatment of lymphocytes from old subjects with IL-2 reversed the impaired production of type-1 cytokines, restored the proliferative response of T cells and rescued from increased apoptosis [33]. Thus the increase in the percentage of lymphocytes expressing intracellular IL-2 may normalize the immune function dependent on this cytokine. Adequate IL-2 secretion is essential in naive CD4^+ ^T cells proliferation in response to T cell receptor (TCR) stimulation and generation of effectors from naïve T lymphocytes pool [34]. Additionally, an antigen-independent process of ageing of T cells occurs because of lowered IL-2 production [35]. Thus, increased IL-2 production as the effect of moderate exercise may contribute in the restoration of naïve T cells pool. The results of investigations performed in other laboratories showed that peripheral blood lymphocytes isolated from active elderly demonstrated higher proliferative response to polyclonal mitogens and higher rates of IL-2, IFN-γ, IL-4 production than elderly sedentary subjects [36]. Experiments on animal models showed that Con A-activated splenocytes from exercised mice produced higher rate of IL-2 [37]. The results of experiments performed with exercised old mice demonstrated the increase of the naïve to memory T cell ration [38].

Our results demonstrate that moderate fitness training may have the potential to increase immune reactions in vivo in elderly women by modulating the expression of cytokines, which normally are depressed with age.

## Conclusions

Moderate, long-term physical activity may modulate the synthesis of certain cytokines, which are important regulators of the immune response, and may ameliorate the status of the immune system in senescence. This amelioration may result in an enhancement of quality of life of older people.

## List of abbreviations

IL – interleukin

## Competing interests

The authors declare that they have no competing interests.

## Authors' Contributions

EK is the author of the physical activity program and conceived of the idea for the study. PS and ND designed the study. ND drafted the manuscript and analyzed the data related to the immune system. EK and PS described the physical training program and data related to Borg scale.

All authors read and approved the final manuscript.

## Pre-publication history

The pre-publication history for this paper can be accessed here:


